# Detection of Alport gene variants in children and young people with persistent haematuria

**DOI:** 10.1007/s00467-024-06538-8

**Published:** 2024-10-01

**Authors:** Natasha Su Lynn Ng, Tomohiko Yamamura, Mohan Shenoy, Helen M. Stuart, Rachel Lennon

**Affiliations:** 1 Department of Paediatric Nephrology, Royal Manchester Children’s Hospital, Manchester University Hospitals NHS Foundation Trust, Oxford Road, Manchester, UK; 2 Wellcome Centre for Cell-Matrix Research, Michael Smith Building, Division of Cell-Matrix Biology and Regenerative Medicine, School of Biological Sciences, Faculty of Biology Medicine and Health, Manchester Academic Health Science Centre, The University of Manchester, Manchester, M13 9PT UK; 3 Manchester Centre for Genomic Medicine, St. Mary’s Hospital, Manchester University Foundation NHS Trust, Manchester, UK; 4 Division of Evolution and Genomic Sciences, School of Biological Sciences, Faculty of Biology, Medicine and Health, Manchester Academic Health Science Centre, University of Manchester, Manchester, UK

**Keywords:** Microscopic haematuria, Genetic kidney disease, Genetic testing

## Abstract

**Background:**

Genetic kidney disease is an important cause of persistent microscopic haematuria in children and young people. We aimed to determine the frequency of variants in the Alport syndrome genes (*COL4A3*, *COL4A4* or *COL4A5*) in individuals under 18 years of age presenting with persistent microscopic haematuria to a single specialist centre in the UK over a 10-year period.

**Methods:**

We conducted a retrospective longitudinal study of individuals referred to a tertiary paediatric nephrology service with persistent microscopic haematuria between April 2012 to 2022.

**Results:**

A total of 224 individuals (female 51.8%) were evaluated with persistent microscopic haematuria of greater than 6 months duration. The age at presentation was 7.5 ± 4.3 years (mean ± SD) with a duration of follow-up of 6.8 ± 4.6 years (mean ± SD). Targeted exome sequencing was performed in 134 individuals and 91 (68%) had a pathogenic or likely pathogenic variant in *COL4A3*, *COL4A4* or *COL4A5*. Only 49.5% of individuals with identified variants had a family history of microscopic haematuria documented and 37.4% (34/91) had additional proteinuria at presentation. *COL4A5* was the most common gene affected and missense variants affecting glycine residues were the most common variant type.

**Conclusion:**

Over two-thirds of children and young people who underwent genetic testing had an identifiable genetic basis for their microscopic haematuria and over half did not have a documented family history. Genetic testing should be part of the evaluation of persistent microscopic haematuria despite a negative family history.

**Graphical abstract:**

**Figure:**
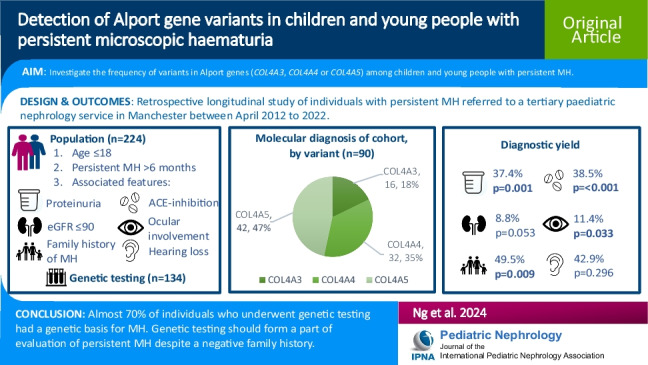
A higher resolution version of the Graphical abstract is available as Supplementary information

**Supplementary Information:**

The online version contains supplementary material available at 10.1007/s00467-024-06538-8.

## Introduction

Asymptomatic, persistent microscopic haematuria (MH) in children and young people has an estimated prevalence of 0.5–2%, varying with definitions of persistent and haematuria [1]. Whilst persistent MH was formerly considered to be a benign finding, it is now a recognized risk factor for chronic kidney disease progression [2, 3]. Furthermore, persistent MH has been linked to an underlying kidney disorder in up to one-third of individuals [4]. Clinical practice guidelines have varied with some recommending follow-up in all cases of MH together with referral to specialist nephrology services depending on the duration of MH and clinical presentation [5, 6].

The evaluation of MH includes a past medical history and family history of kidney disease; screening for hypertension; serum biochemistry for kidney function; complement C3, C4 and immunology screening for glomerulonephritis; urinalysis and urine microscopy, assessment of urinary protein and calcium excretion; and a kidney ultrasound. A kidney biopsy should be considered in the evaluation of MH, particularly if associated with proteinuria or impaired kidney function. However, with isolated MH there is a low diagnostic yield and with the risk of complications associated with a biopsy, it is rarely performed for this indication alone. In the absence of genetic testing, diagnostic evaluation of MH does not lead to a diagnosis in approximately 80% of individuals and longitudinal follow-up for kidney surveillance is typically initiated [6]. However, this strategy can delay diagnosis, and, in some situations, patients are lost to follow-up and later present with advanced chronic kidney disease.

Genetic testing plays an important role in identifying the cause of MH and there is increasing availability of diagnostic panels for cystic kidney disease, glomerular disease, and atypical haemolytic uraemic syndrome. Genetic causes of kidney diseases that initially manifest with persistent MH have been identified and between 30 and 50% of individuals with MH have a familial condition [7, 8]. Variants in *COL4A3, COL4A4* and *COL4A5* are most frequently associated with MH and account for up to 30% of the genetic diagnoses [9]. These genes cause classical Alport syndrome, characterized by kidney, hearing and ocular phenotypes. However, the Alport genes are now associated with a widening clinical spectrum of kidney disease. In a cohort of adults with chronic kidney disease, *COL4A3-5* variants were found in 91 of 3315 (2.7%) individuals. The clinical features were not all typical for Alport syndrome indicating that the frequency of the variants was higher than expected [9]. However, there are limited data on the frequency of variants in the Alport genes amongst children and young people with persistent MH.

In this study, we hypothesized that a significant proportion of children and young people with MH would have a causative variant identified in the Alport genes. We therefore evaluated the frequency of Alport variants associated with persistent MH in individuals presenting to a single centre over a 10-year period.

## Methods

### Study design and participants

In this single centre, retrospective longitudinal study, all outpatient referrals to the tertiary paediatric nephrology service at the Royal Manchester Children’s Hospital between April 2012 and 2022 were assessed. Individuals under 18 years referred for evaluation of persistent MH were included. Persistent MH was defined as MH detected by urine dipstick and confirmed on urine microscopy as ≥ 10 × 10^6^/L red blood cells persisting for > 6 months. Individuals with a subsequent confirmed diagnosis of kidney disease including IgA nephropathy, hypercalciuria, nephrocalcinosis and nephrolithiasis were excluded. Ethical approval was not required as this was a retrospective study with anonymized patient data. Consent for genetic testing was obtained from families or individuals prior to testing.

### Data collection

We collected demographic data including age, sex, ethnicity, age at presentation of MH, age at recognition of *COL4A3-5* variants; clinical features including proteinuria, hypertension, hearing involvement, ocular involvement, chronic kidney disease (CKD), ACE-inhibitor (ACE-I)/angiotensin-receptor blocker (ARB) treatment, family history of haematuria and outcome of genetic testing. Proteinuria was measured on a spot urine sample and defined as a urine protein:creatinine ratio greater than 20 mg/mmol. A family history of haematuria was ascertained by urine dipstick positivity for MH in either or both parents. Chronic kidney disease was defined as eGFR < 90 ml/min/1.73 m^2^. Electronic health records were reviewed to identify ophthalmology and audiology assessments to ascertain hearing and ocular involvement. Hearing involvement was confirmed with abnormal audiometry demonstrating sensorineural hearing loss or use of hearing aids. Ocular involvement was evaluated based on the presence of characteristic ophthalmic signs typical of Alport syndrome such as perimacular flecks or anterior lenticonus. To examine the genetic characteristics of the cohort, the following variant classification was used: frameshift, in-frame deletion, missense — glycine, missense — non-glycine, nonsense, and splicing variants.

### Genetic testing

The indications for targeted exome sequencing differed during the study interval ranging from all individuals evaluated with persistent MH of greater than 6 months duration to the more restricted criteria outlined within the National Genomic Test Directory for haematuria [10]. This currently allows testing in a proband with haematuria when: (1) a first-degree relative with haematuria of unexplained chronic kidney disease, OR (2) electron microscopy evidence of Alport syndrome or thin basement membranes; OR (3) clinical features of Alport syndrome (sensorineural hearing loss, perimacular flecks or anterior lenticonus). In this study, targeted genetic testing using an 8-gene next-generation sequencing panel for haematuria was carried out via the NHS genomic diagnostic laboratory. Specifically, this testing examined for sequence variants, exon deletions and duplications in the *COL4A1*, *COL4A3*, *COL4A4*, *COL4A5*, *MYH9*, *CFHR5* and *NPHS2* genes and for exon deletions and duplications in the *COL4A6* gene. Sequence variants were confirmed by fluorescent bidirectional Sanger sequencing. Sequence variants were evaluated, and pathogenicity of genetic results was assigned based on American College of Medical Genetics and Genomics (ACMG) guidelines [11]. In brief, identified variants were classified as pathogenic, likely pathogenic, variant of uncertain significance, likely benign and benign. Comparison of observed frequencies of variant types in *COL4A3-5* genes was made with manual evaluation of the ClinVar database and the Genome Aggregation Database (gnomADv2.1.1, www.gnomAD.broadinstitute.org) [12, 13].

### Statistical analysis

For comparison of MH duration between children who did or did not have genetic testing a *t*-test was used. Fisher’s exact and chi-square tests were used to examine the association of predictive features of MH including gender, hypertension, hearing loss, ocular involvement, proteinuria, ACE-I/ARB use, family history of haematuria and CKD with diagnostic yield of *COL4A3-5* variants. The Kruskall-Wallis test was used to examine the associations of median age at presentation and median age at diagnosis between the different groups. We performed a logistic regression model to test for association between associated clinical characteristics and the inherited *COL4A3-5* variants. Statistical analysis was performed using SPSS Statistics 25 (IBM Corporation, Armonk, NY, USA).

## Results

### Cohort characteristics

The mean duration of follow-up in this study was 6.8 ± 4.6 years (Table 1). There was a slight female predominance (51.8%) and the age at presentation with persistent MH was 7.5 ± 4.3 years. The duration of MH in children who underwent genetic testing compared to individuals who did not was 2.4 ± 2.3 years and 2.8 ± 3.3 years, respectively. The majority of individuals were White (68.4%), followed by Asian (23.2%), Mixed (3.7%), Other (3.2%) and Black (1.6%). Amongst the associated features, family history of MH was the most common (66%), followed by hearing loss (28.7%). In the full cohort, proteinuria was noted in 18.8% of individuals at presentation. At the latest follow-up, ACE-I or ARB use was recorded in 18.3%, ocular changes in 8.5% and chronic kidney disease in 3.6%. Only 56.3% of individuals with proteinuria at the latest follow-up were prescribed ACE-I or ARB and this likely reflects changing clinical practice over the study interval.

**Table 1:** Clinical characteristics of patient cohort with MH

	Total patient cohort, *n* = 224	Patient cohort who underwent genetic testing, *n* = 134
**Age**		
At presentation, *years*	7.5 ± 4.3	7.7 ± 4.4
At diagnosis, *years*		10.1 ± 4.7
**Sex**	*n* (%)	*n* (%)
Male	108 (48.2)	77 (57.5)
Female	116 (51.8)	57 (42.5)
**Ethnicity**	*n* (%)	*n* (%)
Asian	44/190 (23.2)	20/105 (19)
Black	3/190 (1.6)	1/105 (1)
Mixed	7/190 (3.7)	3/105 (2.9)
White	130/190 (68.4)	75/105 (71.4)
Other	6/190 (3.2)	6/105 (5.7)
**Clinical features**	*n* (%)	*n* (%)
Proteinuria — at presentation	42/224 (18.8)	38/134 (28.4)
ACE-i/ARB	41/224 (18.3)	38/134 (28.4)
Hearing loss	31/108 (28.7)	29/64 (45.3)
Ocular involvement	8/94 (8.5)	8/51 (15.7)
Family history of haematuria	70/106 (66.0)	56/77 (72.7)
Chronic kidney disease	8/224 (3.6)	8/134 (6.0)
**Variant classification**		*n* of variants (%)
Frameshift		4/86 (4.4)
In-frame deletion		4/86(4.4)
Missense — glycine		40/86 (44.4)
Missense — non-glycine		6/86 (6.7)
Non-sense — loss of function		25/86 (27.8)
Splicing		11/86 (12.2)

The number of individuals with no family history of MH or CKD was 36/106 where a family history was documented. Within our cohort, the number of individuals who underwent a kidney biopsy was 10/224 and all 10 had abnormal basement membranes on electron microscopy. The number of individuals with sensorineural hearing loss was 31/109 and the number with ocular findings documented was 8/94.

### Genetic testing identified a high frequency of variants in COL4A3-5

**Fig. 1:**
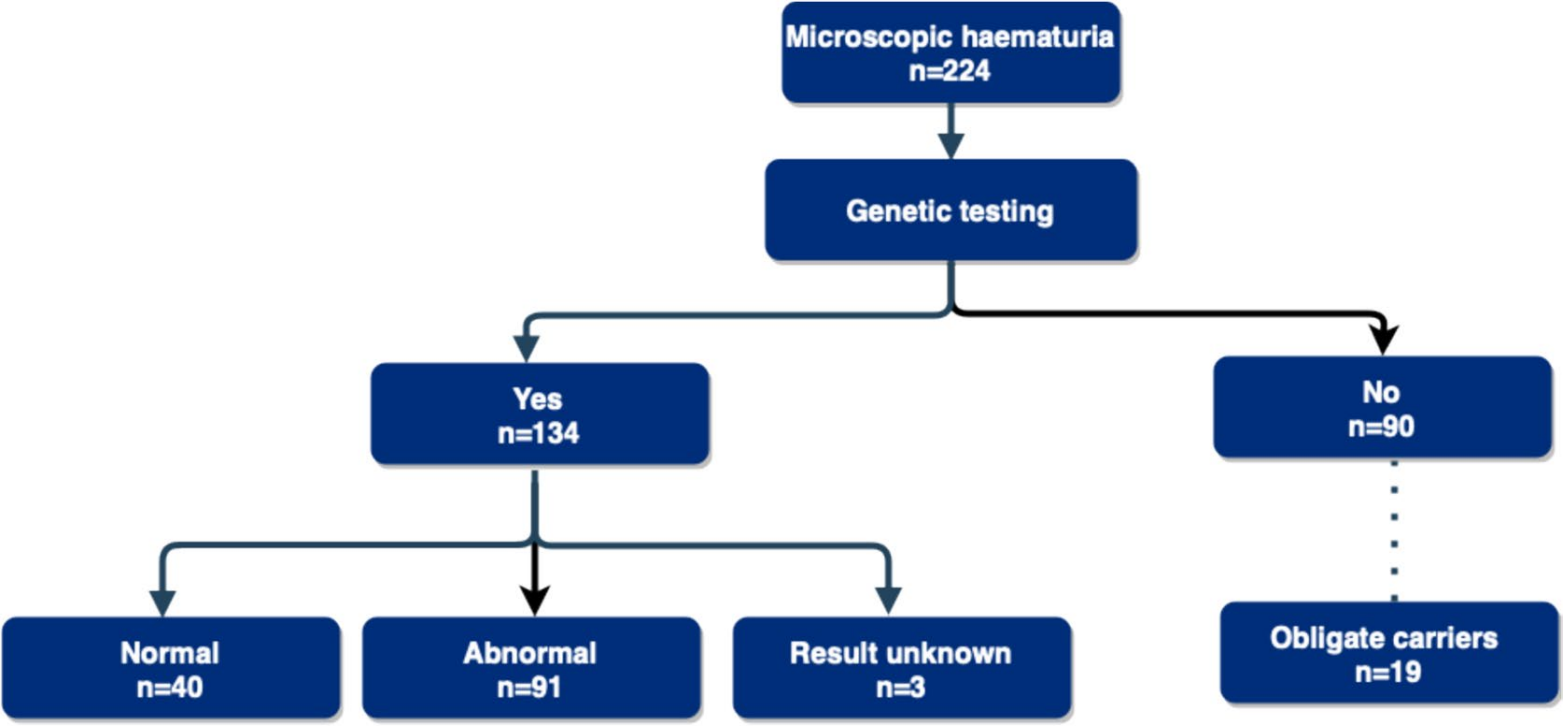
Outcomes of genetic testing for microscopic haematuria. A total of 224 children and young people aged ≤ 18 years with persistent MH were referred over the 10-year period. Targeted exome sequencing was performed in 134 individuals. A positive genetic test was observed in 91/134 individuals (68%) who were identified to have a pathogenic or likely pathogenic variant in *COL4A3*, *A4*, or *A5*. Of the 90 individuals who did not undergo genetic testing, 19 individuals were assumed obligate carriers from affected relatives

A total of 224 individuals aged ≤ 18 years with persistent MH were referred over the 10-year period (Fig. 1). Targeted exome sequencing was performed in 134 individuals. A positive genetic test was observed in 91/134 individuals (68%) who were identified to have a pathogenic or likely pathogenic variant in *COL4A3-5*. Of the 40/134 individuals without variants in *COL4A3-5*, one child was found to have a variant of uncertain significance in *MYH9*. The outcomes of 3 individuals who underwent genetic testing were not available on our electronic patient record system. Amongst the 90 individuals who did not undergo genetic testing, 19 individuals were assumed obligate carriers from affected relatives.

The number of individuals with no family history of MH or CKD documented and who underwent genetic testing was 22/36. Within the cohort, the number of individuals who underwent a kidney biopsy and who also had a genetic testing was 10/10. The number of individuals with sensorineural hearing loss who underwent genetic testing was 29/31, and the number of individuals with ocular findings documented who underwent genetic testing was 8/8.

The number of individuals with no family history of MH or CKD who had a pathogenic or likely pathogenic variant in *COL4A3-5* was 11/36. Within the cohort, the number of individuals with a biopsy who had a pathogenic or likely pathogenic variant in *COL4A3-5* was 7/10. The number of individuals with sensorineural hearing loss who had a pathogenic or likely pathogenic variant in *COL4A3-5* was 29/31, and the number of individuals with ocular findings documented who had a pathogenic or likely pathogenic variant in *COL4A3-5* was 5/8.

### Distribution of genetic variants

**Fig. 2:**
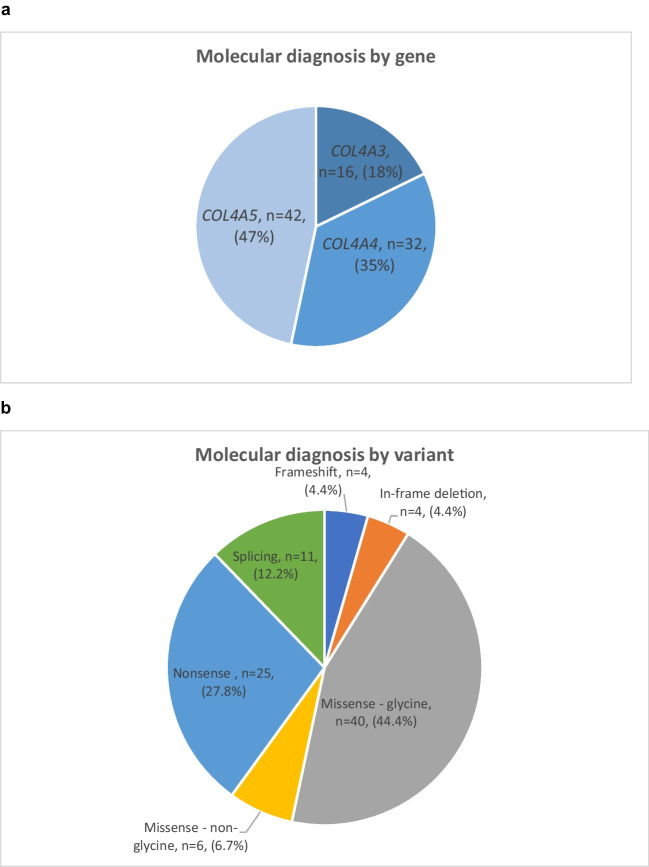
**a** Molecular diagnosis of the whole cohort. A pathogenic or likely pathogenic variant in *COL4A5* was most common, with 42 individuals identified (26 hemizygous males and 16 heterozygous females). Pathogenic or likely pathogenic variants in *COL4A3* were identified in 16 individuals (10 heterozygous, 5 compound heterozygous and 1 homozygous) and pathogenic or likely pathogenic variants in *COL4A4* were identified in 32 individuals (27 heterozygous, 1 compound heterozygous and 4 homozygous). The genetic variant for one individual with autosomal recessive AS was not available in their health records. **b** Variant characteristic for whole cohort. 90 pathogenic or likely pathogenic variants in *COL4A3, A4 or A5* were identified with missense variants affecting glycine residues being most frequently identified in 40 individuals, followed by nonsense variants in 25 individuals, splicing variants in 11 individuals and missense variants affecting non-glycine residues in 6 individuals. Frameshift and in-frame deletion variants were the least commonly observed within our cohort with 4 individuals identified to have each of these variant types

A pathogenic or likely pathogenic variant in *COL4A5* was most common, with 26 hemizygous males and 16 heterozygous females identified (Fig. 2a). Pathogenic or likely pathogenic variants in *COL4A3* were identified in 16 individuals (10 heterozygous, 5 compound heterozygous and 1 homozygous), and pathogenic or likely pathogenic variants in *COL4A4* were identified in 32 individuals (27 heterozygous, 1 compound heterozygous and 4 homozygous). Although confirmed, the genetic variant for one individual with autosomal recessive AS was not available in their health records. Eighty-six pathogenic or likely pathogenic variants in *COL4A3*-*5* were identified with missense variants affecting glycine residues being most frequently identified in 40 individuals, followed by nonsense variants in 25 individuals, splicing variants in 11 individuals and missense variants affecting non-glycine residues in 6 individuals (Fig. 2b). Frameshift and in-frame deletion variants were the least commonly observed within our cohort with 4 individuals identified to have each of these variant types.

### Genotype and phenotype correlation

Factors that were predictive of a positive genetic test included a family history of MH and the presence of proteinuria (Table 2). 45/91 (45.9%) individuals with identified variants in *COL4A3-5* had a family history of MH. This compares to 10/40 (25%) of individuals who had a relevant family history but who did not have a variant identified, and this difference was significant (*p* = 0.009). For proteinuria at presentation, 34/91 (37.4%) individuals had a positive genetic test compared to 4/40 (10%) individuals who did not, and this difference was also significant (*p* = 0.001).

**Table 2:** Analysis of associated predictive features of MH with *COL4A3-COL4A5* variants

Clinical features	Negative Genetic Test, *n* = 40	Positive Genetic Test, *n* = 91	*p*-value
**Sex**	*n* (%)	*n* (%)	0.542
Male	21 (52.5)	53 (58.2)	
Female	19 (47.5)	38 (41.8)	
**Hypertension**			0.452
Yes	2 (5.0)	8 (8.8)	
No	38 (95.0)	83 (91.2)	
**Proteinuria**			0.001
Yes	4 (10.0)	34 (37.4)	
No	36 (90.0)	51(62.6)	
**ACE-I/ARB**			< 0.001
Yes	3 (7.5)	35 (38.5)	
No	37 (92.5)	56 (61.5)	
**Hearing loss**			0.296
Yes	5 (62.5)	24 (42.9)	
No	3 (37.5)	32 (57.1)	
**Ocular involvement**			0.033
Yes	3 (42.9)	5 (11.4)	
No	4 (57.1)	39 (88.6)	
**Family history of haematuria**			0.009
Yes	10 (25.0)	45 (49.5)	
No	30 (75.0)	46 (50.5)	
**Chronic kidney disease**			0.053
Yes	0	8 (8.8)	
No	40 (100)	83 (91.2)	

**Fig. 3:**
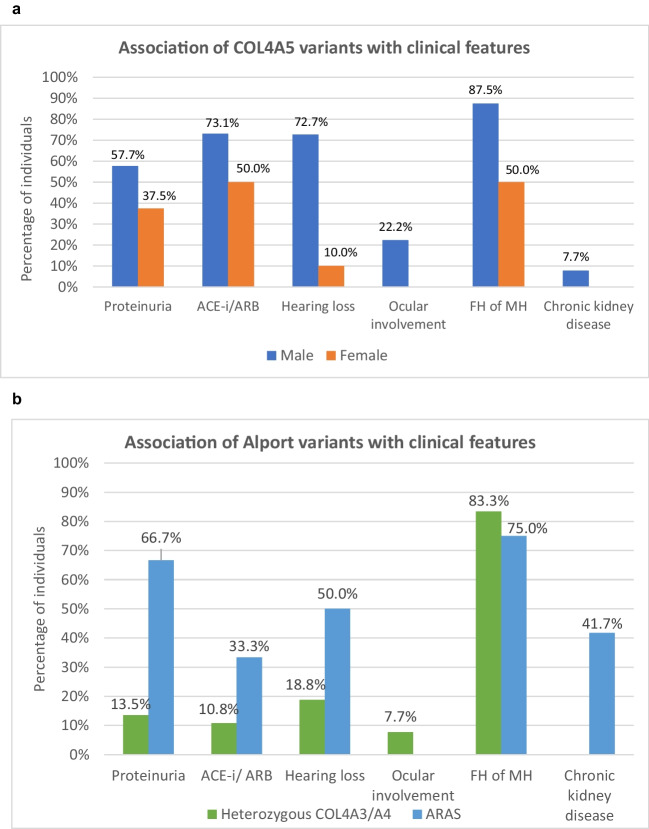
**a** Frequency of clinical features in children with *COL4A5* variants, by sex. Family history of MH (87.5%), use of ACE-I/ ARB (73.1%), hearing loss (72.7%) and proteinuria (57.7%) were amongst clinical features most observed in males. In comparison, a lower frequency of family history of microscopic haematuria (50%), use of ACE-I/ ARB (50%), proteinuria (37.5%) and hearing loss (10%) were observed in females. Ocular involvement (22.2%) and chronic kidney disease (7.7%) were features only observed in males with *COL4A5* variants in our cohort. **b** Distribution of frequency of clinical features in children with heterozygous *COL4A3/A4* variants compared to children with autosomal recessive AS. The frequencies of family history of MH (75%), proteinuria (66.7%), hearing loss (50%), chronic kidney disease (41.7%) and use of ACE-I/ ARB (33.3%) were higher in individuals with autosomal recessive AS compared to individuals with heterozygous *COL4A3/A4* variants. Ocular involvement was observed in one individual (7.7%) with heterozygous *COL4A3/A4* variant and none of the individuals with autosomal recessive AS. Chronic kidney disease was observed in none of the individuals with heterozygous *COL4A3/A4* variants in our cohort

Forty-two individuals (male 61.9%) had a genetic diagnosis of X-linked Alport syndrome (XLAS) due to pathogenic or likely pathogenic variants in *COL4A5*. Amongst the 42 variants in *COL4A5*, missense variants affecting the glycine residue were observed in 24 individuals (57.1%), followed by splicing variants in 8 individuals (19.0%), nonsense (loss of function) variants in 6 individuals (14.3%), frameshift variants in 3 individuals (7.1%) and in-frame deletion variants in one individual (2.4%). The median age at presentation of MH for these individuals was 6.5 (3.5–10.4) years and the median age at diagnosis was 9.3 (6.9–14.1) years. Most individuals had associated features including proteinuria (50%) and family history of haematuria (77.3%). Hearing loss was observed in 53.1%, and ocular changes were reported in 16% of individuals. Hearing loss and ocular changes were more frequently reported in boys with variants in *COL4A5* compared to girls with a frequency of 16/17 (94.1%) for hearing loss and 4/4 (100%) for ocular changes (Supplementary Table 1). The frequency of clinical features in males and females with *COL4A5* variants was variable (Fig. 3a). Family history of MH (87.5%), proteinuria (57.7%), use of ACE-I/ ARB (73.1%) and hearing loss (72.7%) were amongst clinical features most observed in males. In comparison, a lower frequency of family history of MH (50%), use of ACE-I/ ARB (50%), proteinuria (37.5%) and hearing loss (10%) were observed in females. Ocular involvement (22.2%) and chronic kidney disease (7.7%) were features only observed in males with *COL4A5* variants in our cohort.

Twelve individuals (male 75%) had autosomal recessive Alport syndrome (ARAS) due to homozygous or compound heterozygous pathogenic or likely pathogenic variants in *COL4A3* or *COL4A4*. 4/12 individuals had missense variants affecting glycine residues and 4/12 individuals had missense variants affecting non-glycine residues. A further 2 individuals had nonsense variants and one individual each had frameshift and splicing variants. The median age at presentation of MH was 7.5 years (4.1–11.1), and the median age at diagnosis was 9.4 years (7.0–14.3). 8/12 individuals had proteinuria; a family history of MH was reported in 4, and in 3/4 individuals, it was positive; hearing health was reported in 8 with hearing loss detected in 4/8 of individuals and no ocular changes were reported. 5/12 individuals developed chronic kidney disease with an average age of onset of kidney failure at 17.8 years (Supplementary Table 2).

A total of 37 individuals (male 48.6%) had heterozygous pathogenic or likely pathogenic variants in *COL4A3* or *COL4A4*. Of the 36 variants identified, nonsense variants were most common and occurred in 17 individuals (47.2%), followed by 12 individuals (33.3%) with missense variants affecting glycine residues, 3 individuals (8.3%) with in-frame deletion variants and 2 individuals (5.6%) each with missense variants affecting non-glycine residues and splicing variants. The median age at presentation of MH for these individuals was 7.0 (4.0–10.8) years, and the median age at diagnosis was 9.4 (7.0–14.3) years. Family history of haematuria was the most frequently observed clinical feature in 25/30 (83.3%) where the data were available. Other associated features included proteinuria in 5/37 (13.5%) individuals, hearing loss in 3/16 (18.8%) individuals and ocular changes in 1/13 (7.7%) individuals. Hearing loss and ocular changes were observed in three individuals and one child respectively with variants in *COL4A4* (Supplementary Table 3).

The frequencies of proteinuria (75%), family history of MH (75%), hearing loss (50%), chronic kidney disease (41.7%) and use of ACE-I/ ARB (33.3%) were higher in individuals with ARAS compared to individuals with heterozygous *COL4A3/A4* variants. Ocular involvement was observed in one individual (7.7%) with heterozygous *COL4A3/A4* variant, and none of the individuals with ARAS. Chronic kidney disease was observed in none of the individuals with heterozygous *COL4A3/A4* variants in our cohort (Fig. 3b).

Our data also showed that males with XLAS were more likely to present with proteinuria; however, there was no significant difference between individuals with XLAS and individuals with ARAS (*p* = 0.347) (Supplementary Table 4). Individuals with XLAS and ARAS showed a higher incidence of proteinuria at presentation compared to those with heterozygous variants in *COL4A3*/A4 (*p* < 0.001). As expected, individuals with XLAS and ARAS were more likely to have proteinuria and develop CKD compared to individuals with heterozygous variants in *COL4A3/A4* (*p* < 0.001) (Supplementary Table 4). Hearing loss (*p* < 0.001) and family history of MH (*p* = 0.024) were features more likely to be present in males with *COL4A5* variants compared to females with *COL4A5* variants (Supplementary Table 5). A logistic regression model investigating the association between the clinical characteristics and genetic variants of *COL4A3-5* variants showed that proteinuria (*p* < 0.001) and family history of haematuria (*p* < 0.001) were amongst features predictive of a positive genetic test (Supplementary Table 6).

### Comparison of the cohort by the kidney phenotype

57/96 (59.4%) individuals with isolated persistent MH had variants in *COL4A3-5* compared to 34/38 (89.5%) individuals with persistent MH and proteinuria. Individuals with isolated MH had heterozygous *COL4A3/COL4A4* variants (56%) compared to XLAS (69.2%) in individuals with MH and proteinuria and ARAS (62.5%) in individuals with MH, proteinuria and reduced GFR. A family history of haematuria was reported amongst individuals with isolated MH (80%) and those with MH, proteinuria and reduced GFR (100%) compared to individuals with MH and proteinuria (69.2%). Hearing loss was observed most amongst individuals with MH, proteinuria and reduced GFR (85.7%) compared to 50% in those with MH and proteinuria and 31.3% in individuals with isolated MH. Missense variants affecting glycine residues were observed in 37.5% individuals with MH compared to 57.1% with MH and proteinuria and 50% with MH, proteinuria and reduced GFR (Supplementary Table 7).

### Frequency of *COL4A3, A4, A5* variants and comparison with curated variants

In this cohort for *COL4A3*, missense variants were most prevalent accounting for 83.4% of variants (Supplementary Table 8). Similarly, missense variants were most prevalent in *COL4A5* representing 57.1% of the identified variants. This compares to proportions in the ClinVar database where missense variants in *COL4A3* are 28.3% and *COL4A5* are 44.1% of reported variants. For *COL4A4*, the most prevalent alterations observed within our cohort were nonsense (63.3%) variants, including the pathogenic European *COL4A4* founder variant c.2906C > G (p.(Ser969*)). This variant has previously been shown to occur at a frequency of one in 15,608 individuals [14], and it is present in population databases at a relatively low frequency (rs35138315, gnomAD 0.0001%). We observed this founder variant in 18/19 (94.7%) individuals with *COL4A4* variants. We observed a comparable proportion of *COL4A4* missense variants (25.8%) to ClinVar (23.3%). Of note, missense variants affecting the glycine residue were prevalent amongst individuals with variants in *COL4A3*, *COL4A4* or *COL4A5* with a frequency of 10/15 (66.7%), 6/7 (85.7%) and 24/24 (100%), respectively.

## Discussion

This study summarizes the investigation of individuals referred to a tertiary paediatric nephrology service with persistent MH. It is set in the context of a centrally commissioned genomic testing service within the UK’s National Health Service (NHS) which was established in 2018, in the latter half of the study interval.

We found a high detection rate of variants in *COL4A3*, *A4 or A5* among a cohort of paediatric patients with MH of up to 68%. Our detection rates were higher amongst individuals with additional proteinuria (89.5%) compared to individuals with isolated persistent MH without proteinuria (59.4%). This is a rate higher than reported by other studies both in adults and children, although our detection rates amongst individuals with isolated persistent MH without additional proteinuria were comparable to a study which reported diagnostic rates of 58% in children with microscopic haematuria who underwent testing with a limited haematuria panel, comprising *COL4A1*, *COL4A3*, *COL4A4*, *COL4A5* and *MYH9* [15]. The study by Alge et al. however had a lower diagnostic yield of 33% amongst children with isolated persistent MH irrespective of family history of haematuria [16]. The higher detection rate in our study is likely to reflect selection bias from clinical referral and genetic testing. In a separate study examining the diagnostic utility of exome sequencing for CKD, variants in *COL4A3-5* were found in 30% of individuals [9]. Another study in children found a similar diagnostic rate of 28.1% among children with MH, a family history of CKD or deafness and the diagnostic rate was highest at 51.3% among children with MH and a family history of kidney disease [17]. In addition, a study from France combining a cohort of high-risk adults and children with familial MH yielded a diagnostic rate of 82% following targeted testing for *COL4A3*-*5* [18].

In addition, our study showed that the presence of a family history of MH was predictive of a variant in *COL4A3-5* with detection rates of 49.5% compared to 25% in individuals without a family history of MH. Whilst the presence of a family history of MH makes detection of a pathogenic or likely pathogenic *COL4A3-5* variant more likely, a negative family history of MH or absence of extra-renal manifestations cannot reliably exclude variants in Alport genes. Therefore, genetic testing in individuals with persistent MH along with consideration of a kidney biopsy if genetic testing is not diagnostic is a pragmatic approach.

Proteinuria at presentation was also predictive of variants in *COL4A3-5* with detection rates of 37.4% compared to 15% of individuals who did not have proteinuria. This finding supports recent recommendations to extend indications for screening for pathogenic variants in *COL4A3-5* genes beyond classical Alport phenotype of haematuria, kidney failure, family history of haematuria or kidney failure [19]. More recent studies have identified pathogenic *COL4A3-5* variants to be responsible for up to 20% of sporadic or familial adult-onset focal segmental glomerulosclerosis (FSGS) with proteinuria or steroid-resistant nephrotic syndrome as a primary presenting feature [20, 21]. Consequently, testing for variants in *COL4A3-5* should be considered when genetic testing is performed in these patient groups. As our study utilized a targeted gene panel, whole genome sequencing may improve diagnostic yield for some patients [22].

Each of the genes *COL4A3-5* encodes an alpha chain of type IV collagen [23]. These chains consist of a long triple helical domain with repeating Gly-X–Y sequences, where the amino acid glycine is followed by other amino acids at positions X and Y. The repeating sequence determines protein structure, allowing flexibility and unique mechanical properties [24]. As with previous studies, we observed that missense variants affecting glycine residues in the Gly-X–Y repeats were the most common pathogenic type [19]. A 100,000 Genomes Project study where heterozygous pathogenic *COL4A3*/*4* variants were correlated with the risk of MH found an increased risk of MH (*p* = 0.018) with variants that resulted in a glycine substitution with a destabilising amino acid (Arg, Val, Glu, Asp, Trp) [25]. In our cohort, for heterozygous missense pathogenic *COL4A3/4* variants affecting glycine residues, 11/12 (91.7%) individuals had a substitution with a destabilising amino acid.

ACE-I/ARB therapy has been shown to delay onset of kidney failure in individuals with variants in Alport genes. Retrospective studies from Europe and Japan have demonstrated a difference in time to kidney failure of up to 20-plus years in treated patients compared to those who were untreated [26, 27]. A more recent prospective randomized trial in children further demonstrated the beneficial effects of ramipril in reducing the slope of albuminuria and reduction in eGFR between patients who received treatment and those who did not by almost 50% with a good safety profile (hazard ratio, 0.51; 95% CI, 0.12–2.20; *p* = 0.4) [28]. Among our cohort of patients with MH and proteinuria, at their last follow-up, only 56.3% of individuals were receiving either an ACE-I or ARB and this likely represents changes in clinical practice during the study interval. Expert recommendations now propose that ACE-I should be started in children with XLAS or ARAS prior to the onset of proteinuria [28]. Genetic testing of children with MH will help with the early identification of those at risk of progressive CKD and who will benefit from early initiation of treatment with ACE-I.

There are positive implications of genetic testing as part of the diagnostic evaluation of MH. The frequency of kidney surveillance can be set according to the genetic variant and risk of CKD progression, and cascade testing of at-risk relatives is possible with a genetic diagnosis in index cases. One study reported that 39% of patients who had genetic testing had a change in diagnosis, and 59% had a change in clinical management including treatment, follow-up frequency or avoidance of a kidney biopsy [29]. Similar to observations from previous studies, our findings also demonstrate that whilst heterozygous pathogenic or likely pathogenic *COL4A3*/*4* variants are common in individuals with familial MH, affected individuals are less likely to develop CKD or have associated hearing loss or ocular abnormalities [30]. Furthermore, our study also supports a newer understanding about the population frequencies of *COL4A3-5* variants. Heterozygous variants in *COL4A3/4* are more common than previously thought. We found that individuals with XLAS comprised 42/91 (46.2%) individuals with abnormal genetic results, followed by heterozygous variants in *COL4A3/A4* in 37/91 (40.7%) individuals and ARAS in 12/91 (13.2%) individuals. Relative population frequencies found an increased ratio of XLAS to heterozygous variants in *COL4A3/4* of ~ 1:20, whilst ARAS occurred at a frequency of < 1% [30].

There are several limitations to this study. Firstly, due to the retrospective nature of our study, the selection criteria of individuals who underwent genetic testing and those who did not were determined by individual clinical practice. Furthermore, current Genomics England testing criteria for MH state that family history of haematuria is required, and this may have contributed to the underestimation of *COL4A3-5* variants. Secondly, this was a retrospective review of clinical information stored on an electronic health record system and there were missing data. Thirdly, this was a single-centre study within a tertiary service and the diagnostic yield for *COL4A3-5* variants may have been underestimated if investigations were performed at local centres. Lastly, clinical information on self-reported family history of MH and availability of audiology and ophthalmology reports were amongst added limitations that may not have been fully captured within clinical correspondence. In addition, there is incomplete penetrance of hearing and eye phenotypes in Alport syndrome, and the presence of these phenotypes is also influenced by the age at the time of the clinical assessment, and this is likely to be relevant in our cohort. Eye changes are not observed before teenage years and hearing loss is unusual under the age of 5 years.

## Conclusion

Overall, our results confirm the importance of genetic testing for children and young people with MH. Over two-thirds of individuals who underwent testing had a genetic basis for MH. Genetic testing should be part of the diagnostic work-up of children with persistent MH despite a negative family history. Correct and timely diagnosis has implications for clinical management, potential avoidance of invasive diagnostic procedures and allows the identification of family members at risk of having the same genetic variant.

## Supplementary Information

Below is the link to the electronic supplementary material.

Graphical Abstract (PPTX 159 KB)

Supplementary file1 (DOCX 40.1 KB)

## Data Availability

The data collected for this study are available from the corresponding author, upon reasonable request.
