# Supplementary material for: Detection of Alport gene variants in children and young people with persistent haematuria
Source: Pediatr Nephrol. 2024 Oct 1;40(3):719–29. doi: 10.1007/s00467-024-06538-8 (PMC11746956; doi:10.1007/s00467-024-06538-8)
Supplement: Supplementary file 1 — Graphical Abstract (PPTX 159 KB) [file 467_2024_6538_MOESM1_ESM.pptx]

## Slide 1
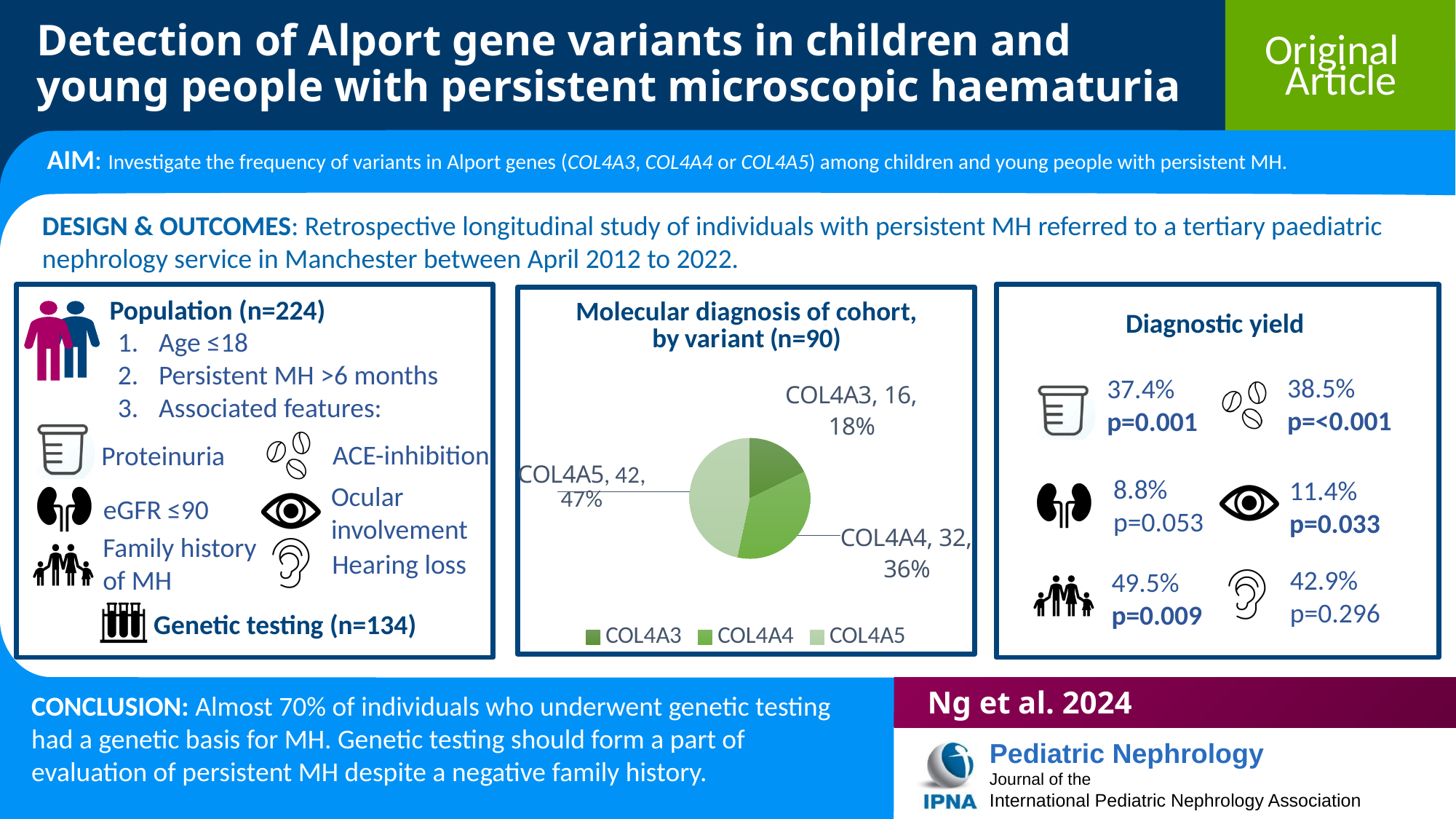

Detection of Alport gene variants in children and young people with persistent microscopic haematuria
AIM: Investigate the frequency of variants in Alport genes (COL4A3, COL4A4 or COL4A5) among children and young people with persistent MH.
DESIGN & OUTCOMES: Retrospective longitudinal study of individuals with persistent MH referred to a tertiary paediatric nephrology service in Manchester between April 2012 to 2022.
Population (n=224)
### Chart: Molecular diagnosis of cohort, by variant (n=90)
| Category | Column1 |
|---|---|
| COL4A3 | 16.0 |
| COL4A4 | 32.0 |
| COL4A5 | 42.0 |
Diagnostic yield
Age ≤18
Persistent MH >6 months
Associated features:
38.5%
p=<0.001
37.4%
p=0.001
ACE-inhibition
Proteinuria
8.8%
p=0.053
11.4%
p=0.033
Ocular involvement
eGFR ≤90
Family history of MH
Hearing loss
42.9%
p=0.296
49.5%
p=0.009
Genetic testing (n=134)
Ng et al. 2024
CONCLUSION: Almost 70% of individuals who underwent genetic testing had a genetic basis for MH. Genetic testing should form a part of evaluation of persistent MH despite a negative family history.
