# Supplementary material for: Detection of Alport gene variants in children and young people with persistent haematuria
Source: Pediatr Nephrol. 2024 Oct 1;40(3):719–29. doi: 10.1007/s00467-024-06538-8 (PMC11746956; doi:10.1007/s00467-024-06538-8)
Supplement: Supplementary file 2 — Supplementary file1 (DOCX 40.1 KB) [file 467_2024_6538_MOESM2_ESM.docx]

| Supplementary Table 1 Clinical characteristics of patient cohort with XLAS (n=42) | | |
| --- | --- | --- |
| Age  At presentation, *years*  At diagnosis, *years* |  | 6.5 (IQR 3.5-10.4)  9.3 (IQR 6.9-14.1) |
| Sex  Male  Female |  | *n* (%)  26 (61.9)  16 (38.1) |
| Ethnicity  Asian  Black  Mixed  White  Other | *n* with data = 32 | *n* (%)  7 (21.9)  0  3 (9.4)  21 (65.6)  1 (3.1) |
| Clinical features  Proteinuria  ACE-i/ ARB  Hearing loss  Ocular changes  FH of haematuria  Chronic kidney disease | *n* with data  42  42  32  25  22  42 | *n* (%)  21 (50)  27 (64.3)  17 (53.1)  4 (16.0)  17 (77.3)  3 (7.1) |
| Clinical features  Proteinuria  ACE-i/ ARB  Hearing loss  Ocular changes  FH of haematuria  Chronic kidney disease | *n* with data  42  42  32  25  22  42 | Male, *n* (%); Female, *n* (%)  15/26 (57.7); 6/16 (37.5)  19/26 (73.1); 8/16 (50.0)  16/22 (72.7); 1/10 (10.0)  4/18 (22.2); 0/7 (0)  14/16 (87.5); 3/6 (50.0)  3/26 (11.5); 0/16 (0) |
| Variant classification  Frameshift  In-frame deletion  Missense – glycine  Missense – non-glycine  Non-sense – loss of function  Splicing |  | *n* of variants (%)  3/42 (7.1)  1/42 (2.4)  24/42 (57.1)  0  6/42 (14.3)  8/42 (19.0) |

| Supplementary Table 2 Clinical characteristics of patient cohort with autosomal recessive AS (n=12) | | |
| --- | --- | --- |
| Age  At presentation, *years*  At diagnosis, *years* |  | 7.2 (IQR 4.1-11.1)  9.4 (IQR 7.0-14.3) |
| Sex  Male  Female |  | *n* (%)  9 (75.0)  3 (25.0) |
| Ethnicity  Asian  Black  Mixed  White  Other | *n* with data = 7 | *n* (%)  1 (14.3)  0  0  6 (85.7)  0 |
| Clinical features  Proteinuria  ACE-i/ ARB  Hearing loss  Ocular changes  FH of haematuria  Chronic kidney disease | *n* with data  12  12  8  6  4  12 | *n* (%)  8 (66.7)  4 (33.3)  4 (50)  0  3 (75)  5 (41.7) |
| Variant classification  Frameshift  In-frame deletion  Missense – glycine  Missense – non-glycine  Non-sense – loss of function  Splicing |  | *n* of variants (%)  1/12 (8.3)  0  4/12 (33.3)  4/12 (33.3)  2/12 (16.8)  1/12 (8.3) |

| Supplementary Table 3 Clinical characteristics of patient cohort with heterozygous *COL4A3/A4* variants (n=37) | | |
| --- | --- | --- |
| Age  Age at presentation, median (IQR), *years*  Age at diagnosis, median (IQR), *years* | | 7.0 (4.0-10.8)  9.4 (7.0-14.3) |
| Sex  Male  Female |  | *n* (%)  18 (48.6)  19 (51.4) |
| Ethnicity  Asian  Black  Mixed  White  Other | *n* with data = 23 | *n* (%)  5 (21.7)  1 (4.3)  0  16 (69.6)  1 (4.3) |
| Clinical features  Proteinuria  ACE-i/ ARB  Hearing loss  Ocular changes  FH of haematuria  Chronic kidney disease | *n* with data  37  37  16  13  30  37 | *n* (%)  5 (13.5)  4 (10.8)  3 (18.8)  1 (7.7)  25 (83.3)  0 |
| Clinical features  Proteinuria  ACE-i/ ARB  Hearing loss  Ocular changes  FH of haematuria  Chronic kidney disease | *n* with data  37  37  16  13  30  37 | *COL4A3, n* (%); *COL4A4*, n (%)  4 (80); 1(20)  2 (50); 2 (50)  0; 3 (100)  0; 1 (100)  5 (20); 20 (80)  0 |
| Variant classification  Frameshift  In-frame deletion  Missense – glycine  Missense – non-glycine  Non-sense – loss of function  Splicing |  | *n* of variants (%)  0  3/36 (8.3)  12/36 (33.3)  2/36 (5.6)  17/36 (47.2)  2/36 (5.6) |

**Supplementary Table 4** Comparison of the clinical characteristics of children with XLAS, ARAS and heterozygous *COL4A3/A4*

|  | XLAS  *n*=42 | ARAS  *n*=12 | Het *COL4A3/A4*  *n*=37 | *P* value  XLAS vs ARAS | *P* value  ARAS vs Het *COL4A3/A4* | *P* value  XLAS vs Het *COL4A3/A4* | *P* value  XLAS vs ARAS vs Het *COL4A3/A4* |
| --- | --- | --- | --- | --- | --- | --- | --- |
| Age at presentation, median (IQR), *years* | 6.5  (3.5-10.4) | 7.6  (3.5-14.4) | 7.2 (4.4-11.3) | 0.216 ^a^ | 0.505 ^a^ | 0.251 ^a^ | 0.183 ^a^ |
| Age at diagnosis, median (IQR), *years* | 9.3  (6.9-14.1) | 10.0  (6.4-15.3) | 9.7  (7.0-14.7) | 0.578 ^a^ | 0.655 ^a^ | 0.241 ^a^ | 0.464 ^a^ |
| Sex  Male, *n* (%) Female, *n* (%) | 26 (61.9) 16 (38.1) | 9 (75.0) 3 (25.0) | 18 (48.6) 19 (51.4) | 0.506 ^b^ | 0.182 ^b^ | 0.264 ^c^ | - |
| Hypertension  Yes, *n* (%) No, *n* (%) | 6 (15) 34 (85) | 2 (16.7) 10 (83.3) | 0 (0) 37 (100) | 0.575 ^b^ | 0.056 ^b^ | **0.027** ^b^ | **0.026**^b^ |
| Proteinuria  Yes, *n* (%) No, *n* (%) | 21 (50) 21 (50) | 8 (66.7) 4 (33.3) | 5 (13.5) 32 (86.5) | 0.347 ^b^ | **<0.001**^b^ | **<0.001** ^c^ | **<0.001** ^b^ |
| ACE-i/ ARB use  Yes, *n* (%) No, *n* (%) | 27 (64.3) 15 (35.7) | 4 (33.3) 8 (66.7) | 4 (10.8) 33 (89.2) | 0.096 ^b^ | 0.088 ^b^ | **<0.001** ^b^ | **<0.001** ^b^ |
| Hearing Loss  Yes, *n* (%)  No, *n* (%) | 17 (53)  15 (47) | 4 (36.4)  7 (63.6) | 3 (15.8)  16 (84.2) | 0.525 ^b^ | 0.051 ^b^ | **0.001** ^b^ | **0.002** ^b^ |
| Ocular changes  Yes, *n* (%) No, *n* (%) | 4 (16) 21 (84) | 0 (0) 6 (100) | 1 (7.7) 12 (92.3) | 0.575 ^b^ | 1.000^b^ | 0.206^b^ | 0.230 ^b^ |
| FH of haematuria  Yes, *n* (%) No, *n* (%) | 17 (77.3) 5 (22.7) | 3 (75) 1 (25) | 25 (83.3) 5 (16.7) | 0.500 ^b^ | **0.017**^b^ | **0.024** ^b^ | **0.011** ^b^ |
| Chronic kidney disease  Yes, *n* (%) No, *n* (%) | 3 (7.1) 39(92.9) | 5 (41.7) 7 (58.3) | - 37 (100) | **0.010** ^b^ | **<0.001**^b^ | 0.243 ^b^ | **<0.001** ^b^ |

^a^ Kruskal-Wallis between groups

^b^ Fisher’s exact test between groups

^c^ Chi-square test between groups

**Supplementary Table 5** Comparison of clinical characteristics of affected COL4A5 males and females

|  | COL4A5 Male  *n*=26 | COL4A5 Female  *n*=16 | *P* value  *COL4A5* male vs *COL4A5* female |
| --- | --- | --- | --- |
| Age at presentation, median (IQR), *years* | 4.4  (2.8-9.8) | 6.3  (2.0-14.0) | 0.887 ^a^ |
| Age at diagnosis, median (IQR), *years* | 8.3  (4.9-10.6) | 12.4  (6.7-16.0) | 0.157 ^a^ |
| Sex  Male, *n* (%) Female, *n* (%) | 26 (100) 0 (0) | 0 (0) 16 (100) | - |
| Hypertension  Yes, *n* (%) No, *n* (%) | 4 (15.4) 22 (84.6) | 2 (12.5) 14 (87.5) | 1.000 ^b^ |
| Proteinuria  Yes, *n* (%) No, *n* (%) | 15 (57.7) 11 (42.3) | 6 (37.5) 10 (62.5) | 0.341 ^c^ |
| ACE-i/ ARB use  Yes, *n* (%) No, *n* (%) | 19 (73.1) 7 (26.9) | 8 (50) 8 (50) | 0.188 ^c^ |
| Hearing Loss  Yes, *n* (%)  No, *n* (%) | 16 (69.6)  7 (30.4) | 1 (9.1)  10 (90.9) | **<0.001** ^b^ |
| Ocular changes  Yes, *n* (%) No, *n* (%) | 4 (21.1) 15 (78.9) | 0 (0) 16 (100) | 0.138 ^b^ |
| FH of haematuria  Yes, *n* (%) No, *n* (%) | 14 (53.8) 12 (46.2) | 3 (18.8) 13 (81.2) | **0.024** ^b^ |
| Chronic kidney disease  Yes, *n* (%) No, *n* (%) | 3 (11.5) 23 (88.5) | - 16 (100) | 0.275 ^b^ |

^a^ Kruskal-Wallis between groups

^b^ Fisher’s exact test between groups

^c^ Chi-square test between groups

**Supplementary Table 6** Logistic regression model investigating the association between clinical characteristics and the inherited genetic variants of *COL4A3, A4* or *A5*

| Characteristic | Odds ratio (95%CI) | *P* value |
| --- | --- | --- |
| Age at presentation | 0.99 (0.98-1.01) | 0.723 |
| Sex (male) | 1.14 (0.45-2.92) | 0.785 |
| Hypertension | 2.90 (0.33-25.69) | 0.339 |
| Proteinuria | 14.57 (4.44 - 47.78) | **<0.001** |
| Hearing loss | 3.51 (0.66 – 18.77) | 0.142 |
| Ocular changes | 7.36 (0.81 – 66.67) | 0.076 |
| FH of haematuria | 6.22 (2.28 – 17.02) | **<0.001** |

| \| Supplementary Table 7 Comparison of clinical characteristics of patient cohort with persistent MH with and without proteinuria \| \| \| \| \| --- \| --- \| --- \| --- \| \|  \| **Persistent MH**  ***n* = 96** \| **Persistent MH with proteinuria**  ***n* = 30** \| **Persistent MH with proteinuria and CKD**  ***n* = 8** \| \| Genetic test outcome  Normal  Abnormal  Unknown \| 36  **57**  3 \| 4  **26**  0 \| 0  **8**  0 \| \| Clinical features  ACE-i/ ARB  Hearing loss  Ocular changes  FH of haematuria \| *n* (%)  13/57 (22.8)  10/32 (31.3)  1/24 (4.2)  32/40 (80) \| *n* (%)  19/26 (73.1)  9/18 (50)  3/15 (20)  9/13 (69.2) \| *n* (%)  3/8 (37.5)  6/7 (85.7)  1/6 (16.7)  3/3 (100) \| \| Clinical phenotype  XLAS  ARAS  Heterozygous *COL4A3/A4* variants \| *n* (%)  21 (36.8)  4 (7)  32 (56) \| *n* (%)  18 (69.2)  3 (11.5)  5 (19.2) \| *n* (%)  3 (37.5)  5 (62.5)  0 \| \| Variant classification  Frameshift  In-frame deletion  Missense – glycine  Missense – non-glycine  Non-sense – loss of function  Splicing \| *n* of variants (%)  1 (1.8)  4 (7.1)  21 (37.5)  4 (7.1)  20 (35.7)  6 (10.7) \| *n* of variants (%)  2 (7.1)  0  16 (57.1)  2 (7.1)  3 (10.7)  5 (17.9) \| *n* of variants (%)  1 (16.7)  0  3 (50)  0  2 (33.3)  0 \| |
| --- | --- | --- | --- | --- | --- | --- | --- | --- | --- | --- | --- | --- | --- | --- | --- | --- | --- | --- | --- | --- | --- | --- | --- | --- |

| Supplementary Table 8 Frequency of variants – a comparison to simple ClinVar | | | | | | |
| --- | --- | --- | --- | --- | --- | --- |
|  | **COL4A3** | | **COL4A4** | | **COL4A5** | |
|  | **ClinVar**  **n=939 (%)** | **Cohort**  **n=18**  **(%)** | **ClinVar**  **n=1062**  **(%)** | **Cohort**  **n=30**  **(%)** | **ClinVar**  **n=1552**  **(%)** | **Cohort**  **n=42**  **(%)** |
| Frameshift | 51  (5.4) | 1  (5.5) | 69  (6.5) | 0 | 216  (13.9) | 3  (7.1) |
| In-frame deletion | 13  (1.4) | 0 | 11  (1.0) | 3  (10) | 30  (1.9) | 1  (2.4) |
| Missense - glycine | 266  (28.3) | 10  (55.6) | 274  (25.8) | 6  (20) | 684  (44.1) | 24  (57.1) |
|  |  |  |  |  |  |  |
| Missense – non-glycine |  | 5  (27.8) |  | 1  (3.3) |  | 0 |
| Non-sense – loss of function | 57  (6.1) | 0 | 49  (4.6) | 19  (63.3) | 107  (6.9) | 6  (14.3) |
| Splicing | 222  (23.6) | 2  (11.1) | 193  (18.2) | 1  (3.3) | 311  (20) | 8  (19) |
